# Synthesis, Characterisation and Biological Activity of Thienoyl- and Furanoyl-Derived Schiff-Base Compounds With 3*d* Metal Ions

**DOI:** 10.1155/MBD.1998.355

**Published:** 1998

## Abstract

Cobalt(ll), copper(ll), nickel(ll) and zinc(ll) metal complexes of 2-carboxybenzylidene thienoylhydrazide HL^1^ and 2-carboxybenzylidene furenoylhydrazide HL^2^ of the type [M(L)_2_.Cl_2_] have been prepared where M = Co(ll) and Ni(ll) and L = HL^1^ and HL^2^ (Fig 1) and characterized by their physical, analytical and spectral studies. The different mode of chelation of the ligands with metals and the effect of these metals on their biological properties is discussed.


### SYNTHESIS, CHARACTERISATION AND BIOLOGICAL ACTIVITY OF THIENOYL- AND FURANOYL-DERIVED SCHIFF-BASE COMPOUNDS WITH 3*d* METAL IONS

[1] Department of Chemistry, Islamia University, Bahawalpur, Pakistan
[2] Department of Chemistry, Washington University, St. Louis, MO 63130, USA

**ABSTRACT**
Cobalt(II), copper(II), nickel(II) and zinc(II) metal complexes of 2-carboxybenzylidene thienoylhydrazide $HL^1$ and 2-carboxybenzylidene furenoylhydrazide $HL^2$ of the type $[M(L)_2.Cl_2]$ have been prepared where M = Co(II) and Ni(II) and L = $HL^1$ and $HL^2$ (Fig 1) and characterized by their physical, analytical and spectral studies. The different mode of chelation of the ligands with metals and the effect of these metals on their biological properties is discussed.

### INTRODUCTION
Previous reports[1-3] on the coordination and biological properties of aroyl hydrazines[4,5] and their aromatic Schiff-bases[6,7], especially those derived from pyridine and pyrazine provide a reasonable model for the mechanism of amine oxidase inhibition[8]. In an effort to extend this program to the hitherto uninvestigated compounds $HL^1$ and $HL^2$ (Figure 1) which contain as many as the potential donor sites and are expected to lead to varied bonding and stereochemical behavior in the complexes, we wish to report the results of our investigations on the synthesis and structural studies of Co(II) and Ni(II) complexes with the title Schiff bases, and their biological properties in the present paper.

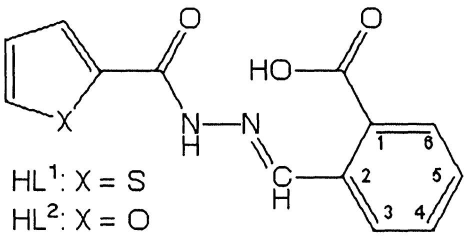

Fig 1 Structure of the Schiff bases $HL^1$ and $HL^2$

### EXPERIMENTAL
### Material and Methods
All chemicals and solvents used were of Analar grade. All the metals were used as their metal(II) chloride salts. 2-Thiophenecarboxylic hydrazide, 2-furoic hydrazide and 2-carboxybezyldehyde used for the preparation of the Schiff bases were obtained from Aldrich Chemical Company. IR, $^1$H NMR and $^{13}$C NMR spectra were recorded on Philips Analytical PU 9800 FTIR and Brucker 250 MHz instruments. UV- Visible spectra were obtained on a Hitachi U-2000 double-beam spectrophotometer. Conductance of the metal complexes was determined in DMF at $10^{-3}$ dilution on a YSI-32 model conductometer. Magnetic measurements were done on solid complexes using the Gouy method. The synthesized Schiff bases and their metal chelates were analyzed for C, H and N by microanalytical techniques and the metal contents in the chelates were estimated by the standard methods. Melting points were recorded on a Gallenkamp apparatus and are uncorrected. The antibacterial studies were carried out with the help of the Department of Pathology, Quaid-e-Azam Medical College, Bahawalpur, Pakistan.

### Preparation of the Schiff bases
**2-Carboxybenzylidene thienoylhydrazide ($HL^1$)**
An ethanol solution of 2-carboxybenzaldehyde (0.01 M, 20 mL) was added to a mechanically stirred ethanol solution of 2-thiophenecarboxylic hydrazide (0.01 M, 15 mL) respectively. Then 2-3 drops of conc $H_2SO_4$ were added in it and mixture refluxed for 1 h. The reaction mixture was then cooled in an ice-bath, which immediately gave a precipitated product. The product thus obtained was filtered, washed thoroughly with ethanol, then with ether and dried. The precipitated product was crystallized from aqueous ethanol to give $HL^1$ (75 %). The same method was used for the preparation of $HL^2$ (68 %) except 2-furanecarboxylic hydrazide was used instead of 2-thiophenecarboxylic hydrazide

**Preparation of Metal Complexes**

An ethanol solution of appropriate metal(II) chloride (1 mmol, 10 mL) was added to a stirred hot ethanol solution of the respective Schiff base (2 mmol, 30 mL). The resulting mixture was refluxed for 2 h. The solution was then cooled, filtered and reduced to nearly half its volume. Adding ether in excess and immediately precipitating effected the metal complexes. The precipitates were filtered, washed with ethanol, then with ether and dried. These were crystallized from hot aqueous ethanol to give metal complexes (1-8).

**Antibacterial Studies**

**Preparation of Discs.**

The Schiff base/complex (30 (g) in DMF (0.01mL) was applied on a paper disc, [prepared from blotting paper (3 mm diameter)] with the help of a micropipette. The discs were left in an incubator for 48 h at $37^{o}$ C and then applied on the bacteria grown agar plates.

**Preparation of Agar Plates.**

Minimal agar was used for the growth of specific bacterial species. For the preparation of agar plates for *Escherichia coli*, MacConkey agar (50 g), obtained from Merck Chemical Company, was suspended in freshly distilled water (1 L). It was allowed to soak for 15 minutes and then boiled on a water bath until the agar was completely dissolved. The mixture was autoclaved for 15 minutes at $120^{o}$ C and then poured into previously washed and sterilized Petri dishes and stored at $40^{o}$ C for inoculation.

**Procedure of Inoculation.**

Inoculation was done with the help of a platinum wire loop which was made red hot in a flame, cooled and then used for the application of bacterial strains.

**Application of Discs.**

A sterilized forceps was used for the application of paper disc on the already inoculated agar plates. When the discs were applied, they were incubated at $37^{o}$ C for 24 h. The zone of inhibition was then measured (in diameter) around the disc.

**RESULTS AND DISCUSSIONS**

The Schiff bases were prepared by following the method as reported[5-7] earlier. The structural determination of these synthesized ligands was done with the help of their spectral and analytical data.

The IR spectra of the free Schiff bases (Table 1) show some characteristic bands at 3220, 1735, 1680 and 1635 $cm^{-1}$ assigned respectively to (-NH), (-C=O), (-COOH) and (-C=N) frequencies. The absence of bands at 3345 and 1725 $cm^{-1}$ due to amine ($-NH_2$) and carboxyldehyde (H-C=O) provided a strong evidence[9-11] for the formation of Schiff bases $HL^1$ and $HL^2$. Also, $^1H$ NMR, $^{13}C$ NMR spectral data (Table 2) of the title Schiff bases showed all the expected protons and carbons on comparison of these values with the reported one[12]. Moreover, their CHN percentage also confirmed the molecular formulae and their structures.

**Table 1 Physical, Spectral and Analytical Data of Schiff bases**

| No | Mol.Formula | M.P (°C) | IR ($cm^{-1}$) | Calc(Found)% C H N |
|---|---|---|---|---|
| $HL^1$ | $C_{13}H_9N_2O_3S$ [273.2] | 163 | 3220(s,NH),1735(vs,C=O), 1680(s,$CO_2H$),1635(s,C=N) | 57.2 3.3 10.2 (57.4) (3.1) (10.5) |
| $HL^2$ | $C_{13}H_9N_2O_4$ [261.1] | 177 | 3220(s,NH),1738(vs,C=O), 1685(s,$CO_2H$),1635(s,C=N) | 59.8 3.4 10.7 (60.0) (3.5) (10.9) |

s = strong, vs = very strong

The reaction of Schiff bases with metal(II) chloride salts yielded complexes of 1 : 2 (M : L) stoichiometric composition. All complexes are air/moisture stable solids. They are insoluble in common organic solvents such as chloroform, ethanol, acetone, dichloromethane and benzene. They are however, partially soluble in water and completely soluble in DMF and

DMSO. The conductivity measurements (18-25 $ohm^{-1}$ $cm^2$ $mol^{-1}$) of these complexes in DMF indicated[21,22] them to be non-electrolytes[13,14].

**Table 2 $^1$H NMR and $^{13}$C NMR Data of Schiff bases**

| No | $^1$H NMR (ppm) | $^{13}$C NMR (ppm) |
|---|---|---|
| $HL^1$ | 6.2(dd,1H,heterocyclic),5.8(s,1H, CH=N),6.4(d,2H,heterocyclic),6.8-6.9(m,1H,H-3),7.4-7.5(m,2H,H-4,5), 7.8-7.9(m,1H,H-6),8.3(s,1H,NH), 9.2(s,1H,$CO_2$H). | 106.3(C-heterocyclic),116.4(C-heterocyclic), 122.2(C-2),127.6(C-5),128.7(C-6),132.7(C-4), 137.8(C-1),142.9(C-heterocyclic),152.3(C=N), 153.1(C,*ipso*-heterocyclic),188.4 (C=O),194.3($CO_2$H). |
| $HL^2$ | 6.1(dd,1H,heterocyclic),5.7(s,1H, CH=N),6.5(d,2H,heterocyclic), 6.7-6.8(m,1H,H-3),7.4-7.5(m,2H,H-4,5), 7.8-7.9(m,1H,H-6), 8.3(s,1H,NH),9.2(s,1H, $CO_2$H). | 107.1(C-heterocyclic),115.2(C-heterocyclic), 122.2(C-2),127.6(C-5),128.7(C-6),132.7(C-4), 137.8(C-1),143.7(C-heterocyclic), 152.3(C=N),153.3(C,*ipso*-heterocyclic), 188.4 (C=O),194.3($CO_2$H). |

The important infrared spectral bands of the metal chelates are discussed in Table 3. The band due to the azomethine (C=N) linkage appeared at 1635 $cm^{-1}$ in the case of all free Schiff bases. A shift to lower frequency of this band (~15-20 $cm^{-1}$) is observed in the case of its metal chelates, indicating coordination through the azomethine nitrogen.

**Table 3 Physical, Spectral and Analytical Data of the Complexes**

| Complex/ Mol.Formula | M.P ($^0$C) | B.M ($\mu_{eff}$) | IR ($cm^{-1}$) | $\lambda_{max}$ ($cm^{-1}$) | Calc(Found)% C H N |
|---|---|---|---|---|---|
| 1 $[Co(HL^1)_2Cl_2]$ $C_{26}H_{14}CoN_4O_6S_2$ [672.2] | 271-273 | 3.85 | 3220,1715,1670, 1615,510,405 | 9045,15215, 27210 | 46.5 2.1 8.3 (46.8)(1.9)(8.2) |
| 2 $[Co(HL^2)_2Cl_2]$ $C_{26}H_{14}CoN_4O_8$ [648.1] | 285-287 | 4.27 | 3220,1720,1675, 1620,515,410 | 8555,15345, 26600 | .48.2 2.2 8.6 (48.4)(2.5)(8.5) |
| 3 $[Ni(HL^1)_2Cl_2]$ $C_{26}H_{14}NiN_4O_6S_2$ [672.0] | 256-258 | 3.04 | 3220,1725,1672, 1622,515,405 | 10512,17533, 28375 | 46.5 2.1 8.3 (46.8)(1.8)(8.3) |
| 4 $[Ni(HL^2)_2Cl_2]$ $C_{26}H_{14}NiN_4O_8$ [647.9] | 267-269 | 2.85 | 3220,1720,1670, 1625,515,410 | 9918,16865, 28950 | 48.2 2.2 8.6 (48.5)(2.1)(8.8) |

The amide (NH) band appearing at 3220 $cm^{-1}$ in the free Schiff base remains unaffected in the metal complexes, which clearly indicates that the imine nitrogen is not taking part in the coordination. The band appearing at 1735 $cm^{-1}$ suffered a negative (~ 25 $cm^{-1}$) shift indicating coordination through the carbonyl oxygen atom of carbonyl group. The carboxylic (COO) group stretching vibrations were found at ~1680 $cm^{-1}$; these are also shifted to lower frequency (~ 8-10 $cm^{-1}$) in the case of the metal complexes, showing coordination through the carboxylic group. The coordination of the metal through the azomethine nitrogen and oxygen donor sites of the ligand was further confirmed by the appearance of the new bands in the regions 405-450 $cm^{-1}$ and 455-510 $cm^{-1}$, which could be assigned[15] to the respective M-N and M-O frequencies.

**Magnetic Moments and UV-Visible Spectra**

The magnetic moment and electronic spectral data of the cobalt(II) and nickel(II) complexes were calculated from the corrected magnetic susceptibility values (Table 3) and suggested[36] an octahedral geometry for all the complexes around the metal ion. The room temperature magnetic susceptibility measurements (Table 3) for the solid complexes lie within the range expected for their octahedral geometry. Three unpaired electrons per Co(II) ion ($\mu_{eff}$ = 3.85-4.27 B.M) and two unpaired electrons per Ni(II) ion ($\mu_{eff}$ = 2.85-3.04 B.M) suggested[16,17] octahedral geometry for Co(II) and Ni(II) complexes. The magnetic susceptibilities have, in octahedral geometry for Co(II) and Ni(II) complexes. The magnetic susceptibilities have, in general, lower values than the expected moments probably due to antiferromagnetism which arises via a superexchange mechanism.

The UV-Visible spectra of the cobalt(II) chelates showed three bands observed at 8555-9045 $cm^{-1}$, 15345-15215 $cm^{-1}$ and 26600-27210 $cm^{-1}$ in its electronic spectra which may be assigned to $^4T_1g \rightarrow {}^4T_2g$ (F), $^4T_1g \rightarrow {}^4A_2g$ (F) and $^4T_1g \rightarrow {}^4T_1g$ (P) transitions, respectively, and are suggestive[18,19] of its idealized octahedral geometry around the cobalt ion. The nickel(II) chelates showed also three bands observed at 9918-10512 $cm^{-1}$, 16865-17533 $cm^{-1}$ and 28375-28950 $cm^{-1}$ due to spin-allowed transitions from $^3A_2g \rightarrow {}^3T_2g$ (F),$^3A_2g \rightarrow {}^3A_1g$ (F) and $^3A_2g \rightarrow {}^3T_1g$ (P), respectively, in an octahedral environment[20].

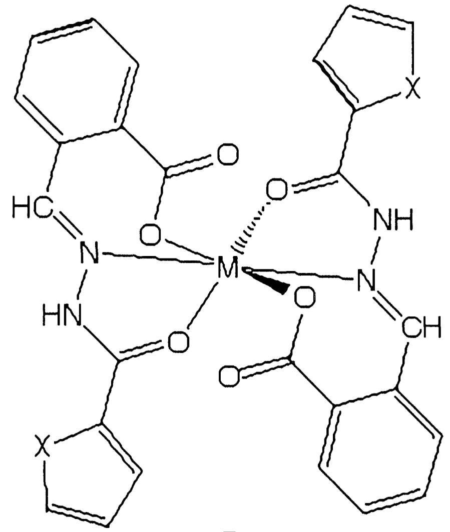

F

igure 2 Proposed Structure of Metal(II) chelate

In light of the above observations, it is suggested that all the metal chelates show an octahedral geometry (Fig 2) in which the two ligand molecules act as tridentate around the metal atom, accommodating themselves in such a way that a stable chelate ring is formed.

**Table 4 Antibacterial Activity Data**

| Ligand/Chelate | M i c r o b i a l S | | p e c i e s |
|---|---|---|---|
| | a | b | c |
| $L^1$ | ++ | + | + |
| $L^2$ | + | ++ | - |
| 1 | ++++ | ++ | ++ |
| 2 | +++ | +++ | +++ |
| 3 | +++ | +++ | +++ |
| 4 | +++ | +++ | +++ |

a = *Escherichia coli*, b = *Pseudomonas aeruginosa*,
c = *Staphylococcus aureus*
Inhibition zone diameter mm (% inhibition): +, 6-10 (27-45 %); ++, 10-14 (45-64 %); +++, 14-18 (64-82 %); ++++, 18-22 (82-100 %). Percent inhibition values are relative to inhibition zone (22 mm) of the most active compound with 100 % inhibition.

Antibacterial Studies

The title Schiff bases, in comparison to their metal complexes, were screened against the bacterial species *Staphylococcus aureus, Escherichia coli, Klebsiella pneumonae* and *Pseudomonas aeruginosa* in order to determine the effect of metal ions on the individual antibacterial activity of metal chelates.

The compounds were tested at a concentration of 30 (g/0.01 mL in DMF solution using paper disc diffusion method. The results of these studies reproduced in Table 4 describe that all the Schiff bases are biologically active and their metal complexes show more significant antibacterial activity against one or more bacterial species than the uncomplexed ligands. It therefore, confirms that chelation tend to make the ligands acting more powerful and potent bactericidal. At this stage it is, however, not clear that why the metal ions increase the activity on complexation. But, it is assumed that the number of bonding sites and chelation reduces considerably the polarity of the metal ion in the complexes due mainly to the partial sharing of its positive charge with the donor group. The possible electron delocalization over the chelate ring system inturn increases the hydrophobic character of the metal chelate thus favouring its permeation through lipoid layer of microorganism.

**Acknowledgement**
We are grateful to the Department of Pathology, Qaid-e-Azam Medical College, Bahawalpur, for the help in undertaking the antibacterial studies.

**References**

1 R. C. Aggarwal, N. K. Singh and R. P. Singh, *Inorg. Chem. Acta*, **20**, 279 (1981).
2 B. Singh, R. N. Singh and R. C. Aggarwal, *Synth. React. Inorg. Met-Org. Chem.*, **14**, 815 (1984).
3 Z. H. Chohan and A. Rauf, *Metal-Based Drugs*, **3**, 211 (1996).
4 Z. H. Chohan and S. K. A. Sherazi, *J. Chem. Soc. Pak.*, **19**, 196 (1997),
5 Z. H. Chohan and A. Rauf, *Synth. React. Inorg. Met-Org. Chem.*, **26**, 591(1996)
6 Z. H. Chohan and S. K. A. Sherazi, *Metal-Based Drugs*, **4**, 69 (1997).
7 Z. H. Chohan and S. K. A. Sherazi, *Metal-Based Drugs*, **4**, 65 (1997).
8 M. F. Iskander, S. E. Zayan, M. A. Khalifa and L. El-Sayed, *J. Inorg. Nucl. Chem.*, **36**, 556 (1974).
9 R. Micholson and G. J. Suttan, *Aust. J. Chem.*, **22**, 373 (1969).
10 B. B. Knal and K. B. Pandeya, *J. Inorg. Nucl. Chem.*, **40**, 1035 (1977).
11 M. P. Teotia, D. K. Rastogi and W. Malik, *Inorg. Chim. Acta*, **7**, 339 (1973).
12 D. H. Williams and I. Fleming, "Spectroscopic Methods in Organic Chemistry", 4th Ed, Mc Graw Hill, London, 1989.
13 M. Shallary, M. M. Moustafa and M. M. Bekheit, *J. Inorg. Nucl. Chem.*, **41**, 267 (1979).
14 W. J. Geary, *Coord. Chem. Rev.*, **7**, 81 (1971).
15 K. Nakamoto, "Inorganic and Raman Spectra of Inorganic and Coordination Compounds", J. Wiley, New York, 1978.
16 C. J. Balhausen, "An Introduction to Ligand Field", Mc Graw Hill, New York, NY, 1962.
17 M. D. Glick and R. L. Lintvedt, *Prog. Inorg. Chem.*, **21**, 233 (1976).
18 R. L. Carlin, "Transition Metal Chemistry", Vol 1, Marcel Decker, New York, 1965.
19 A. B. P. Lever, "Inorganic Electronic Spectroscopy", Elsevier, Amsterdam, 1984.
20 D. W. Meek, R. S. Drago and T. S. Piper, *Inorg. Chem.*, **1**, 285 (1962).

**Received: October 21, 1998 - Accepted: November 11, 1998 - Received in revised camera-ready format: November 26, 1998**

